# CD56-positive diffuse large B-cell lymphoma: comprehensive analysis of clinical, pathological, and molecular characteristics with literature review

**DOI:** 10.2478/raon-2023-0016

**Published:** 2023-03-21

**Authors:** Gorana Gasljevic, Lucka Boltezar, Srdjan Novakovic, Vita Setrajcic-Dragos, Barbara Jezersek-Novakovic, Veronika Kloboves-Prevodnik

**Affiliations:** Institute of Oncology Ljubljana, Ljubljana, Slovenia

**Keywords:** diffuse large B-cell lymphoma not otherwise specified, CD56, immunohistochemistry, genetic profiling, prognosis

## Abstract

**Background:**

Diffuse large B-cell lymphoma (DLBCL) is the most common non-Hodgkin lymphoma. The expression of CD56 in DLBCL is highly unusual. Little is known about its incidence and clinical importance. So far, no genetic profiling was performed in CD56 positive DLBCL.

**Patients and methods:**

Tissue microarrays have been constructed, sectioned, and stained by H&E and immunohistochemistry for 229 patients with DLBCL diagnosed 2008–2017. For CD56 positive cases, clinical data was collected including age at diagnosis, stage of the disease, International Prognostic Index (IPI) score, treatment scheme and number of chemotherapy cycles, radiation therapy, treatment outcome, and possible relapse of the disease. Overall survival (OS) and progression-free survival (PFS) were calculated. For four patients, RNA was extracted and targeted RNA (cDNA) sequencing of 125 genes was performed with the Archer FusionPlex Lymphoma kit.

**Results:**

CD56 expression was found in 7 cases (3%). The intensity of expression varied from weak to moderate focal, to very intensive and diffuse. All patients had *de novo* DLBCL. The median age at the time of diagnosis was 54.5 years. Five of them were women and 2 males. According to the Hans algorithm, 6 patients had the germinal centre B cells (GBC) type and one non-GBC (activated B-cell [ABC]) type, double expressor. Genetic profiling of four patients according to Schmitz's classification showed that 1 case was of the BN2 subtype, 1 of EZB subtype, 2 were unclassified. The six treated patients reached a complete response and did not experience progression of the disease during the median follow-up period of 80.5 months.

**Conclusions:**

We report on one of the largest series of CD56+DLBCL with detailed clinicopathological data and for the first time described genetical findings in a limited number of patients. Our results show that CD56 expression is rare, but seems to be present in prognostic favourable subtypes of DLBCL not otherwise specified (NOS) as tested by immunohistochemical or genetic profiling.

## Introduction

CD56, also known as the neural cell adhesion molecule (NCAM), is a member of the immunoglobulin superfamily that plays important functional roles during nervous system development, differentiation, and immune surveillance. In addition to neurons and glial cells, CD56 is normally also expressed in neuroendocrine tissues and some cells of the hematopoietic system like NK cells and activated T lymphocytes.^1^ In the hematopathology service, it is mainly used as a marker of NK cells and their neoplastic counterparts. Its aberrant expression is useful as a proof of clonal plasma cell proliferation, while it can also be used as prognostic marker in plasmacytoma, as well as in acute myeloid leukemia (AML) and acute lymphoblastic leukaemia (ALL).^2,3,4,5^

Diffuse large B-cell lymphoma (DLBCL) is the most common lymphoma, representing approximately one third of all non-Hodgkin lymphomas.^2^ Cases of DLBCL that do not fit the distinctive clinical presentation, tissue morphology, neoplastic cell phenotype, and/or pathogen-associated criteria of other subtypes of DLBCL are termed “DLBCL not otherwise specified (DLBCL NOS)’ and represent 80–85% of all DLBCL cases.^2^ The WHO 2016 classification of hematopoietic neoplasms^2^ requires that the neoplastic cells in DLBCL NOS be further defined based on whether they are derived from germinal centre B cells or activated B-cells as identified by gene expression profiling (GEP) or are germinal centre B cells (GBC) or non-GBC as identified by immunohistochemical (IHC) analyses. In general, DLBCL NOS is an aggressive disease with an overall long-term survival rate in patients treated with standard chemotherapy regimens of ~60%.^7,8^ Patients with activated B-cell (ABC) DLBCL and non-GBC variants have significantly worse prognoses than patients with the GBC variant.^6^ Expression of markers in DLBCL NOS neoplastic cells that have clinical significance as prognostic or predictive factors include CD5, MYC, BCL2, BCL6, CD20, CD19, CD22, CD30, PD-L1, and PD-L2.^2,6^ For example, 5–10% of DLBCL NOS cases express CD5 and have a very poor prognosis that is not improved by even aggressive treatment regimens, while the expression of CD30 represents a favourable prognostic indicator.^2^

Very little is known about the incidence and clinical importance of CD56 expression in DLBCL. In the last 30 years, the literature has only a few case reports or small series of CD56+ DLBCL with conflicting results on its importance.^10,11,12,13,14,15,16,17,18^ It could have a prognostic value; however, since new target drugs are becoming available and among them is also anti-CD56 antibody, CD56 could serve as a potential target for the treatment of patients who do not respond to standard therapeutic schemes.

The purpose of this study was to evaluate CD56 expression in DLBCL in our series, to estimate its relationship to epidemiological factors, to roughly estimate its value as a prognostic marker, and to describe, for the first time the molecular findings in a subset of cases.

## Patients and methods

### Specimens

Data bases of the Department of Pathology Institute of Oncology Ljubljana (IOL) have been searched for all cases of DLBCL diagnosed between 2008 and 2017. Only the cases in which appropriate amount of material was present that could allow the construction of tissue microarrays (229) have been chosen for the study. Tissue microarrays have been constructed, sectioned, and stained by H&E and immunohistochemistry for the Hans algorithm as previously described.^19^ Also, for the cases that were CD56 positive, flow cytometric and/or immunocytochemical staining results and data were retrieved and re-analysed from the database of the Department of Cytopathology.

### Patients

For selected patients, clinical data was collected including age at diagnosis, stage of the disease, IPI score, treatment scheme and number of cycles, potential radiation therapy, outcome and possible relapse of the disease were also noted. Overall survival (OS) and progression-free survival (PFS) were calculated. Subjects were censored at their last visit to the IOL and for those who finished follow-up at IOL, a vital status from the Cancer Registry of the Republic of Slovenia. All procedures followed in this evaluation were in accordance with the ethical standards of the responsible committee on human experimentation (Ethical Committee of Institute of Oncology Ljubljana, approval number: ERIDKESOPKR-23 and the Ethical Committee of the Republic of Slovenia, approval number: 58/02/15) and the Helsinki Declaration of 1975, as revised in 2000.

### Immunohistochemistry

3–4 μm thick, formalin-fixed paraffin-embedded sections of constructed TMAs were used for immunohistochemical staining with the monoclonal antibody CD56. Staining was performed on the Ventana Benchmark platform using the MRQ 42 clone (Cell Marque) in dilution 1:200.

### Flow cytometric analysis and immunocytochemistry

The preparation of FNAB (fine needle aspiration biopsy) lymph node sample, cell counting, sample preparations for flow cytometric immunophenotyping, acquisition of cells with flow cytometer and measurement result analysis were performed as previously described.^20^ Monoclonal antibodies against CD45, CD19, CD20, CD3, CD10, CD5, CD23, FMC7, κ and λ LCs (BD Biosciences, New Jersey, U.S.) were used. The samples were acquired using a four-colour flow cytometer FACSCalibur (BD Biosciences, New Jersey, U.S.), a six-colour flow cytometer FACSCanto II (BD Biosciences, New Jersey, U.S.) or a ten-colour FACSCanto X (BD Biosciences, New Jersey, U.S.). The measurement results were analysed using CellQuest (BD Biosciences, New Jersey, U.S.) or BD FACSDiva software (BD Biosciences, New Jersey, U.S.). For immunocytochemical staining, methanol and Delaunay-fixed cytospines were prepared. Stainings were carried out on the Ventana Benchmark Ultra platform using antibodies against CD56, CK AE1/AE3, CK18 (DAKO), CD20 (Cell Marque, Rocklin, California, U.S.), synaptophysin (Termo Scientific, Waltham, Massachusetts, U.S.), CD3 and TTF-1 (Leica Biosystems, Nussloch, Germany).

### Molecular analysis – NGS sequencing

RNA was extracted from 4 paraffin-embedded tissue samples was extracted using the MagMAX^TM^ FFPE DNA/RNA Ultra Kit (ThermoFisher, Waltham, MA, USA). Samples were treated with DNase, during the extraction process. Targeted RNA (cDNA) sequencing of 125 genes was performed with the Archer FusionPlex Lymphoma kit (Invitae-ArcherDX, San Francisco, CA, USA). The final NGS library was quantified using the KAPA Library Quantification Kit (KAPA Biosystems, Merck, Ljubljana, Slovenia) and pair-end sequenced on a MiSeqDx system (Illumina, San Diego, CA, USA). The trimmed FASTQ file was uploaded to Archer Analysis software Version 6.0.3.2, which performed variant and fusion calls along with the determination of cell of origin (ABC or GCB). Variants were considered true positive if the frequency of the variant allele was above 10%, with minimum coverage of 100x.^20^ All variants reported in GnomAD were excluded. Fusions were considered true positive if the fusion event was covered with a minimum of 5 unique reads and the percentage of reads supporting the event was above 10%.^21,22^

### Statistical analysis

For numeric and demographic variables descriptive statistics were used (median, range, standard deviation, percentage). Overall survival and progression-free survival were calculated using the Kaplan-Meier method. Statistical analyses were performed using IBM SPSS Statistics, version 26.

## Results

**FIGURE 1.**
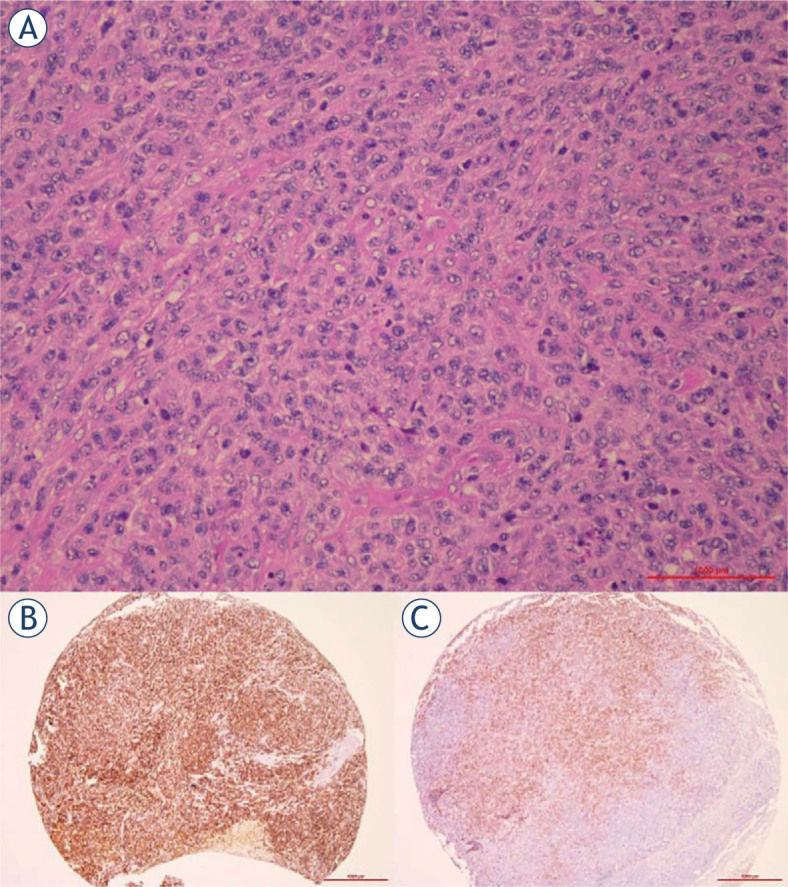
**(A)** Morphology of diffuse large B-cell lymphoma (DLBCL), CD56+; H&E, 20x; **(B)** Strong expression of CD56 in DLBCL not otherwise specified (NOS) (tissue microarray), 4x; **(C)** weak to moderate CD56 expression in DLBCL NOS (tissue microarray), 4x.

Among 229 DLBCL, NOS cases included in the study, CD56 expression was found in 7 cases (3%). The intensity of CD56 expression varied from moderate focal to very intensive and diffuse positive reaction (Figure 1). Reanalysis of the five cases in which fine needle aspiration biopsy (FNAB) of the lymph node was performed prior to surgical biopsy and histological examination showed that CD56 was not included in routine flow-cytometry work-out. There was only one case^23^ (case 1 in Table 1) in which immunocytochemistry for CD56 was stained since tumour cells showed co-expression of cytokeratin and the diagnosis of metastatic neuroendocrine carcinoma has been made.

**TABLE 1.** Clinicopathological characteristics of patients with CD56 positive diffuse large B-cell lymphoma (DLBCL), review of the literature with our series

**Publication**	**Coun**	**No of pat**	**Case No**	**Sex Age**	GC type**	**Non-GC type**	**LN**	**Extranodal disease and site**	**Clinical stage**	**IPI**	**LDH**	**Surg**	**CT and No. of cycles**	**RT**	**Response**	**FU**
Kern 1993^23^	USA	1	1	NA	NA	NA	NA	NA	NA	NA	NA	NA	NA	NA	NA	NA
Muroi 1998^17^	Jap	2	1	M,49	Yes		Yes	Liver, Spleen, Pericard. Ef. Liver	NA	NA	NA	No	CHOP, NAx	No	PR?	NA
			2	F,62		Yes	Yes		NA	NA	NA	No	CHOP, NAx	No	PR?	NA
Sekita 1999^18^	Jap	1	1	M,16	Yes		Yes		I	NA	NA	No	CHOP, 6x	No	CR	10 m
Hammer 1998^15^	USA	4	1	M,51	NA	NA	Yes	Stomach	NA	NA	NA		NA	NA	NA	NA
			2	M,69	NA	NA	No		NA	NA	NA		NA	NA	NA	NA
			3	M,76	NA	NA	Yes		NA	NA	NA		NA	NA	NA	NA
			4	M,54	NA	NA	Yes		NA	NA	NA		NA	NA	NA	NA
Otsuka 2004^14^	Jap	2	1	NA	Yes		NA	NA	NA	NA	NA	NA	NA	NA	NA	NA
			2	NA	Yes		NA	NA	NA	NA	NA	NA	NA	NA	NA	NA
Weisberger *2006^11^			1	M,41	Yes	Yes	No	Ileocecal valve	NA	NA	NA	NA	NA	NA	NA	NA
	USA	10	2	M,52	Yes		Yes	SpineAbdomen Brain	NA	NA	NA	NA	NA	NA	NA	NA
			3	M,54	Yes		Yes		NA	NA	NA	NA	NA	NA	NA	NA
			4	M,83	Yes		No		NA	NA	NA	NA	NA	NA	NA	NA
			5	M,49	Yes		Yes		NA	NA	NA	NA	NA	NA	NA	NA
			6	F,57	Yes		No		NA	NA	NA	NA	NA	NA	NA	NA
			7	F,69	Yes		Yes		NA	NA	NA	NA	NA	NA	NA	NA
			8	M,77	Yes		Yes		NA	NA	NA	NA	NA	NA	NA	NA
			9	M,84	Yes		Yes		NA	NA	NA	NA	NA	NA	NA	NA
			10	M,77			No		NA	NA	NA	NA	NA	NA	NA	NA
Isobe 2007^13^	Jap	3	1	M,80	Yes		Yes	Ascites	NA	NA	NA	No	THP-COP, 3x	No	NR	DOD
			2	F,87	Yes		No	Ileum	NA	NA	NA	Yes	No	No	CR	22 m
			3	M,73	Yes		Yes	Ileum	NA	NA	NA	Yes	R-CHOP, 6x	No	CR	22 m
Chen 2010^16^	Ch	1	1	NA	NA	NA	NA	NA	NA		NA	NA	NA	NA	NA	NA
Gomyo 2010^9^	Jap	7	1	M,29		Yes	Yes	Spleen	IIIB	HI	↑	No	R-CHOP, aPBSCT	No	CR	A, 24 m
			2	F,60		Yes	Yes	WR	IIA	L	N	No	R-CHOP 3x	Yes	CR	A, 50 m
			3	F,22	Yes		No	WR	IA	L	N	No	CHOP 3x	Yes	CR	A, 57 m
			4	M,64		Yes	Yes	Pl. Ef, Adr. gl, Submand. gl	IIIA	H	↑	No	CHOP 5x	No	CR	D, 4 m
			5	M,63	Yes		No	Nasal cavity	IA	L	N	No	RCHOP 3x	Yes	CR	(pneumonia)
			6	M,50	Yes		No	Intra-extradural mass	IA	L	N	No	Res+CHOP 4x	Ye	RCR	A, 43 m
			7	F,45	Yes		No	Subcutis	IVA	HI	↑	No	R-CHOP 8x	sNo		A, 70 mA, 5 m
Stacchini 2012^12^	It	5	1	M,72	Yes		Yes	Spleen, Stomach, Pancr.	NA	NA	NA	NA	NA	NA	NA	NA
			2	M,15	Yes		Yes	Stomach, Liver	NA	NA	NA	NA	NA	NA	NA	NA
			3	M,71	Yes		No	Nasopharynx	NA	NA	NA	NA	NA	NA	NA	NA
			4	M,60					NA	NA	NA	NA	NA	NA	NA	NA
			5	M,21	Yes	Yes	No		NA	NA	NA	NA	NA	NA	NA	AWD 12m
Gu 2013^10^	SK	1	1	F,5		Yes		WR	I		N	Yes	COPAD, 6x	No	CR	NA
Liu 2020^8^	Ch	1	1	M,14	Yes, DH		Yes	Nasopharynx	IV	NA	↑	No	CTX+CP	No	CR	NA
													R-Hyper-CVAD AB			
													R-DA-EPOCH, 6x			
													IT DM+CTB,4x			
Gasljevic 2022	Slo	7	1	F,56	Yes		Yes	Skeletal muscle	IA	0	N	Yes	R-CHOP, 3x	No	CR	A, 63 m
			2	F,51	Yes		Yes	Small bowel	IA	0	N	No	R-CHOP, 3x	No	CR	A, 73 m
			3	M,57	Yes				IIA	0	↑	No	R-CHOP, 6x	No	CR	A, 55 m
			4	M,56		Yes, DE		Spleen, Liver, Adrenal gland	IVB	0	↑	No	R-EPOCH,6x +IT,2x	No	CR	A, 40 m
			5	F,53	Yes		Yes		IIA	3	N	Yes	CHOP, 3x	Yes	CR	A, 62 m
			6	F,30		Yes	Yes		IVB	3	↑	No	R-CHOP,8x	Yes	CR	A, 182 m
			7	F,79	NA	NA	Yes		IVB	5	↑	No	No	No	NA	DOD

A = alive; aPBSCT = autologous peripheral blood stem cell transplantation; AWD = alive with disease; Ch = China; CHOP = cyclophosphamide, doxorubicin hydrocloride, vincristine sulfat, prednisone; Coun = country; CP = prednisone; CR = complete response; CT = chemotherapy; CTX = cyclophosphamide; D = dead; DA-EPOCH = etoposide, doxorubicin, vindesine, dexamethasone, cyclophosphamide; DE = double expressor; DOD = dead of disease; F = female; FU = follow-up; Gl = gland; Hyper-CVAD AB = A: cyclophosphamide, vindesine, liposomal doxorubicin, dexamethason, B: methotrexate, cytarabine; IPI = International Prognostic Index; It = Italy; IT = intratechal; Jap = Japan; LN = lymph nodes; M = male; m = months; N = normal; NA = not available; NR = no response; Pancr = pancreas; Pl. E = pleural effusion; PR = partial response; R = rituximab; res = resection; RT = radiotherapy; SK = South Korea; Slo = Slovenia; Submand = submandibular; THP-COP = pirarubicin, cyclophosphamide, vincristine sulfat, prednisone; WR = Waldeyers ring

* only histologically proven cases are considered

** on the basis of the CD10 positivity

All patients had *de novo* DLBCL. The median age at the time of diagnosis was 54.5 years (range 30–57). Five of them were women and 2 males. Five patients were diagnosed with DLBCL, GC type, 2 with DLBCL non-GC (ABC) type, one being a double expressor (DE). One patient refused staging and treatment and died shortly after being diagnosed and was therefore excluded from survival analysis.

Among the six patients who received treatment, three patients were in clinical stage 1, one in stage 2 while two were in clinical stage 4. Only patients in clinical stage 4 had constitutional symptoms. Four patients had disease localised in the lymph nodes while two of them also had extranodal infiltrates – one in the pectoral muscles and the other in the renal fascia and small bowel. Three patients had elevated LDH levels, in fact, both patients in clinical stage 4B and one in stage 2A. Those patients in stage 4 had the IPI score 3 and others had the IPI score 0.

Three patients underwent surgical procedure and were later treated with adjuvant 3 cycles of CHOP (cyclophosphamide, doxorubicin, vincristine, prednisone) and R-CHOP (rituximab, cyclophosphamide, doxorubicin, vincristine, prednisone). Other 3 patients were treated with 6 or 8 cycles of R-CHOP. Two patients were also treated with adjuvant radiotherapy after completion of systemic treatment. The patient with non-GC type DE of DLBCL was treated with 6 cycles of R-EPOCH (rituximab, etoposide, cyclophosphamide, doxorubicin, vincristine, prednisone) together with 2 doses of intrathecally administered methotrexate and cytosine arabinoside for central nervous system prophylaxis.

The 6 treated patients reached a complete response and did not experience progression of the disease during the follow-up period, meaning that 5-year PFS and OS are 100%. Median follow-up was 80.5 months (range 42–197).

The clinicopathological characteristics of our cohort together with all cases reported in the literature are shown in Table 1. Genetic profiling of 4 patients was performed as described in *Patients and methods*, and the results are presented in Table 2.

**TABLE 2.** Genetic profile of CD56 positive diffuse large B-cell lymphoma (DLBCL) samples

**Case number in Table 2**	**COO IHC**	**COO AFPL**	**fusion**	**variants**			**VAF (%)**	**variant classiification**	**Schmitz et al., 2018^32^ classification**
				gene	nucleotide change	amino acid change			
**1**	GCB	GCB	ND	RANBP1	NM_002882.3:c.23A>G	NP_002873.1:p.(His8Arg)	13,7	Uncertain significance	unclassified
**2**	GCB	GCB	ND	ND	ND	ND	ND	ND	unclassified
**3**	GCB	GCB	ND	CD79B	NM_000626.2:c.587A>T	NP_000617.1:p.(Tyr196Phe)	49,0	Pathogenic	EZB
				CD79B	NM_000626.2:c.568A>G	NP_000617.1:p.(Met190Val)	50,1	Uncertain significance	
				EZH2	NM_001203247.1:c.1922A>G	NP_001190176.1:p.(Tyr641Cys)	53,7	Pathogenic	
				MYD88	NM_001172567.1:c.656C>G	NP_001166038.1:p.(Ser219Cys)	37,7	Uncertain significance	
				SH3BP5	NM_004844.4:c.460G>A	NP_004835.2:p.(Ala154Thr)	19,3	Uncertain significance	
**4**	ABC	ABC	IGH-BCL6	CD79B	NM_000626.2:c.587A>C	NP_000617.1:p.(Tyr196Ser)	25,9	Pathogenic	BN2
				SH3BP5	NM_004844.4:c.460G>A	NP_004835.2:p.(Ala154Thr)	12,6	Uncertain significance	

AFPL = Archer SusionPlex lymphoma; COO= cell of origin; IHC = immunohistochemical analyses; ND = not detected; VAF = variant allele freqency;

## Discussion

CD56 expression in DLBCL NOS is very rare. Its incidence is reported to be 0.5 to 7% of DLBCLs, but is actually unknown since CD56 is generally not included in the immunohistochemical or flow cytometric panel for the diagnosis of DLBCL.^10,11,12,13,14,15,16,17,18^ In our series of patients with DLBCL NOS expression of CD56 was present in 3% of patients and varied in intensity from weak to very strong and diffuse. In one of those cases, that phenomenon resulted in an incorrect diagnosis of lymph node metastasis of the neuroendocrine tumour. In fact, in the general pathology service the main use of CD56 is to prove neuroblastoma and neuroendocrine differentiation in tumours of different origin while in hematopathology service it is used as a marker of NK cells, as a proof of clonal plasma cell proliferation, and as a prognostic marker in plasmacytoma, acute myeloid leukemia (AML), and acute lymphoblastic leukemia (ALL).^2,3,4,5^ Since neuroendocrine carcinomas could be unevenly and weakly positive or even negative for cytokeratins^24^, it is of the greatest importance for the pathologist to be aware that strong expression of CD56 could be present also in some entities that are by definition not CD56 positive.

Throughout the papers published so far, there has been much speculation about this phenomenon with regard to its expression in special clinicopathological settings and its possible prognostic value. From an epidemiological point of view, some authors^9^ suggested that it could be related to racial and/or geographical factors since, at the time of the publication of the paper, almost 50% of all reported cases were reported from Japan. Thorough analysis of all the cases with available information shows that 18 out of 45 cases (40%) have arisen in the population of far east (Japan, Korea, China; Table 1), while 27 (60%) were reported in the western population, Caucasians mainly (USA, Italy, Slovenia; Table 1). These results suggest that CD56+DLBCL is not related to racial / ethnic factors opposite to some other CD56 positive lymphoproliferative diseases such as NK/T cell lymphoma, nasal type.^2^ The age distribution is very wide with cases described in paediatric/adolescent population as well as in the older patient most of the patients being in 6–7^th^ decade of life. In our series, the vast majority of patients were middle aged, in the beginning of the sixth decade. The distribution of gender showed that among the far east patients, somewhat higher number of men are reported (6 female *vs.* 9 males; for 3 cases there is no information about gender) while in the western world there is a predominance of males (7 females *vs.* 19 males; 26% *vs.* 74%). However, our series shows contradictory results in which most patients (70%) are women, so it can be assumed that the higher incidence reported in males so far could be only a mere coincidence.

There are two main biologically distinct molecular subtypes of DLBCL: GCB and ABC. ABC DLBCL is associated with substantially worse outcomes when treated with standard chemoimmunotherapy. Based on gene expression studies, Hans *et al.*^25^ developed an algorithm to discriminate GBC from non-GBC types in regard to immunohistochemical expression of CD10, bcl6 and MUM1 with cutoff of 30%. In addition to GCB and ABC subtypes, double-hit lymphomas and double-expressor lymphomas, which overexpress myc and bcl2 protein, are aggressive DLBCLs and are also associated with a poor prognosis. On the basis of immunohistochemical results, a few authors^9,11,12,13^ found a relation of CD56 expression to DLBCL of GBC origin. Of the 45 summarized cases, for 8 cases there was no information about immunophenotype. Twenty-eight out of 36 (76%) were of GBC type and the remaining 24% were of non-GBC (ABC) type. One reported case^8^ was double hit lymphoma with translocations of *MYC* and *bcl-6,* while in our series one DLBCL of non-GC (ABC) type DLBCL showed so-called double expressor profile with expression of bcl2 and myc protein expression being > 30%. Somewhat lower percentage of GBC types are reported in Eastern patients compared to the Western (10/15 and 17/21 or 75% *vs.* 81%). This finding could be related to the previously recognized and reported lower frequency of the DLBCL GBC subtype in Asian countries.^26^

In addition, it has been suggested that CD56 expression in DLBCL could be related to a more frequent extranodal presentation associated to the adhesive properties of CD56.^9,11^ In neural cells, it mediates cell-to-cell adhesion by CD56 molecules of adjacent cells binding together.^27^ It may be involved in homophilic adhesion for NK and T cells due to the C2-set Ig regions and fibronectin regions within its extracellular domain.^28^ However, its function with respect to B-cell ontogeny is unclear. The expression of CD56 has been detected in a human pluripotent stem cell.^28^ A subset of very early precursor B cells has the innate capacity for CD56 expression that is down-regulated and extinguished later in differentiation. It has been shown that lymphomagenesis is a stepwise process progression of which is enabled by accumulation of genetic events.^8^ In follicular or mantle cell lymphoma, for example^30^, first events such as t(14,18) and t(11,14) namely, do occur in progenitor B cells. Drawing parallels to this, we could assume that CD56+ DLBCL could arise from the precursor B-cell that, for whatever reason, did not down-regulate CD56 expression and then collected additional mutations that resulted in lymphoma development. Some authors^9,11,12,13^ underlined frequent extranodal infiltrates in CD56+DLBCL with spleen, stomach, ileum, and nasal cavity being most frequently involved. Of 40 cases with available information, 16 (40%) presented with isolated lymphadenopathy while 24 (60%) had extranodal infiltrates with or without lymphadenopathy (14 *vs.* 10). Four of our patients presented with isolated lymphadenopathy while two had extranodal disease, which is concordant with majority of our patients having limited stage disease and were therefore treated adjuvantly after surgery.

The expression of CD 56 can be used as a prognostic marker in certain hematopathological entities; it can predict the occurrence of brain infiltration in ALL^5^, the aggressiveness of multiple myeloma^3^, and relapsed AML.^4^ So far, its prognostic importance in DLBCL has not been confirmed. All of our patients achieved complete remission, and remained in remission which can be at least partially attributed to low IPI scores and low clinical stages; however, two patients with clinical stage 4 also achieved and maintained complete remission. None of our patients had a high IPI score of 4 or 5 which are known to have the lowest survival.^31^ In most of them, DLBCL was of GCB subtype, which also carry a better prognosis.^24^

Schmitz *et al.*^32^ classified DLBCL cases according to genetic findings into 4 categories, namely MCD (based on the cooccurrence of *MYD88*^L265P^ and *CD79B* mutations), BN2 (based on *bcl6* fusions and *NOTCH2* mutations), N1 (based on *NOTCH1* mutations) and the EZB group (based on *EZH2* mutations and *bcl2* translocations). These subtypes differed phenotypically and in response to immunochemotherapy, with favourable survival in the BN2 and EZB groups. Genetic profiling of four patients from our series according to Schmitz classification^32^, showed that 1 case was of BN2 subtype, one belongs to the EZB group, while two were unclassified. Although data are limited and demand testing in larger cohorts of patients, so far it can be concluded that CD56 expression is more often present in cases of DLBCL NOS with prognostically favourable genetical findings.

CD56 is expressed in some aggressive tumour types such as small lung cell carcinoma and neuroblastoma. To date, it has been used as a target molecule for antibody-based immunotherapy in phase I and II clinical trials for small cell lung carcinoma^33^; a favourable safety profile has been demonstrated. That led to the development of CAR-T therapy directed against CD56 in neuroblastoma. In the xenograft neuroblastoma model, anti-CD56 therapy led to the tumour burden control but had only modest effect on survival.^34^ More studies are needed in regard to neuroblastoma therapy and other CD56 positive tumours but CD56 could eventually serve as a potential target for the treatment of CD56+ DLBCL patients who do not respond to the standard therapeutic schemes.

In conclusion, here we report one of the largest series of CD56+DLBCL with detailed clinicopathological data and for the first time described genetic findings in a limited number of patients. Our results show that CD56 expression is rare but seems to be present in prognostic favourable subtypes of DLBCL NOS as tested by immunohistochemical or genetic profiling.
